# Influence of dentistry professionals and oral health assistance protocols on intensive care unit nursing staff. A survey study

**DOI:** 10.5935/0103-507X.20170049

**Published:** 2017

**Authors:** Davi Francisco Casa Blum, Jéssica Munaretto, Fernando Martins Baeder, Jussara Gomez, Cristine Pilati Pileggi Castro, Álvaro Della Bona

**Affiliations:** 1 Faculdade de Odontologia, Universidade de Passo Fundo - Passo Fundo (RS), Brazil.; 2 Santa Casa de Misericórida de Porto Alegre - Porto Alegre (RS), Brazil.; 3 Universidade Cruzeiro do Sul - São Paulo (SP), Brazil.; 4 Hospital São Vicente de Paulo - Passo Fundo (RS), Brazil.

## INTRODUCTION

Patients admitted to intensive care units (ICU) often lack oral health assistance,^(1-3)^ with a direct influence on oral health problems related to higher morbidity and mortality. Poor oral health can lead to clinical problems such as locally spreading infections, respiratory tract infections, higher costs of ICU admissions, and higher use of medications such as antibiotics, which can result in the establishment of bacterial resistance and opportunistic infections.^(3-8)^

Considering the value of oral health in preventing complications for ICU patients, it is important to implement oral healthcare protocols in the ICU. This study aimed to evaluate the influence of oral health care protocols, the routine activity of dental professionals, the oral healthcare knowledge of ICU staff and the methods used to provide this care to ICU patients. We tested the hypothesis that oral care protocols and training positively affect oral healthcare practice in the ICU.

## METHODS

This study was approved by the local Ethics Committee of the *Universidade de Passo Fundo* under number 1.879.807 and was performed in accordance with the ethical standards of the Declaration of Helsinki. This cross-sectional descriptive survey study used a self-administered questionnaire given to 231 staff people from 9 ICUs of three Southern Brazilian hospitals. One ICU was in a private hospital, one in a philanthropic hospital and 7 in public hospitals. This study was conducted from March to August 2015.

The self-administered questionnaire had objective (closed-ended) answers and used a 5-point Likert scale and frequency demarcation of oral care procedures. The ICU nursing staff (nurses and technicians) received a language-adapted questionnaire based on the work of Binkley et al.,^(2)^ which focuses on the perception of the professional towards the importance of dentistry in the ICU, oral hygiene practice, staff training, healthcare protocols and dentistry professional routine in the ICU.

Data were subjected to descriptive statistical analysis using Statistical Package for Social Science (SPSS) version 20 (IBM). Descriptive frequency analysis was used to describe quantitative and qualitative data and the Spearman correlation test was used to analyze the 5-point Likert scale questions. P-values <5% were considered significant.

## RESULTS

Of the 231 participants, 182 were technicians and 49 were nurses. Table 1 summarizes the study population characteristics.

**Table 1:** Characteristics of the study participants (intensive care unit staff)

Participants	N	Age (Years-old ± SD)	ICU work time (Years ± SD)
Nurses	49	34.4 ± 7.2	7.2 ± 6.4
Technicians	182	34.3 ± 8.5	5.5 ± 6.2

ICU - intensive care unit; SD - standard deviation. No significant difference found.

The vast majority (99.6%) of the participants agreed on the importance of oral care for ICU patients, and 88.3% of the staff agreed that oral health problems are common in intensive care.

Regarding oral hygiene, 32% of the staff answered that this is an unpleasant task to perform for ICU patients, and 69.3% of the staff had difficulties when performing the task. However, 22.1% of the staff did not receive proper training to perform oral hygiene tasks with ICU patients. Most often (87%), materials and instruments are available for the task, and only 19.5% of the staff stated that there is not enough time to perform oral hygiene tasks for ICU patients.

About a quarter (27.7%) of the staff did not agree on the existence of an adequate oral health protocol for ICU patients, and when an oral problem occurred, only 65.4% of the staff knew how to proceed. Finally, 52.8% of the staff reported the absence of an oral health professional (dentist) to evaluate oral health issues in ICU patients.

The use of oral hygiene materials by the ICU staff varied. Most of the staff (74.1%) never used foam swabs. Oral rinses were commonly and frequently used for oral hygiene of ICU patients. A great variability was found for the use of toothbrush and toothpaste. Table 2 summarizes use frequency of oral hygiene instruments and materials.

**Table 2:** Frequency of use of oral hygiene materials and instruments

	3 hours - 4 hours (%)	8 hours - 12 hours (%)	24 hours or less (%)	Never used (%)
Foam swab	11.7	8.7	5.6	74.1
Oral rinse	48.2	29.1	19.6	3.0
Toothbrush	30.5	40.6	9.6	19.3
Toothpaste	26.5	28.5	10.2	34.7

There was a moderate correlation between professionals who found oral hygiene to be an unpleasant task and those who tended to find this task to be difficult (r_s_ = 0.42, p < 0.001). The lack of an adequate oral health protocol seemed to have a very weak correlation (r_s_ = 0.13, p < 0.05) with professionals perceiving these hygiene tasks to be difficult. The unavailability of materials and lack of sufficient time also influenced the perceived difficulty of performing oral hygiene tasks (r= 0.18, p < 0.05; r = 0.25, p < 0.001 respectively).

The absence of an adequate oral assistance protocol and the absence of training programs were moderately correlated with the staff's inability to solve oral health problems (r = 0.47, p < 0.001; r = 0.43, p < 0.001, respectively). The presence of an oral health professional (dentist) responsible for evaluating oral health issues in ICU patients weakly correlated with staff training, ensuring oral health protocols and increasing staff knowledge regarding oral health problems (r = 0.32, p < 0.001; r = 0.31, p < 0.001; r = 0.25, p < 0.001, respectively).

## DISCUSSION

Questionnaires are an important tool for evaluating and quantifying the habits, procedures, needs and expectations of ICU staff. Binkley et al. evaluated the oral healthcare provided in ICUs in the United States using a survey method. The authors found that the oral care protocols were not uniform and suggested that evidence-based protocols could improve the quality of care and provide more consistent healthcare.^(2)^ Another study evaluated nurses' knowledge, attitudes and practices with questionnaires regarding oral hygiene in hospitals and suggested that most nurses practice some kind of oral health protocols, although these were not standardized among institutions,^(9)^ a finding that aligned with our results. Protocol implementation can also play a role in practice, and active participation of the nursing staff is recommended to produce better results in protocol compliance.^(8)^ Without training, adequate access to materials, and motivation, oral healthcare quality in the ICU is compromised.^(2,8-12)^

The presence of a dentistry professional helps to maintain compliance with the oral healthcare protocols by ensuring that the staff facing difficulties during patient care receive assistance. It is also important to note the association between adequate staff training and the presence of a dentistry professional in the ICU routine. These results are consistent with other studies.^(11-13)^

We found high variability in the use of oral hygiene materials, even among professionals in a single ICU. This variability points to the lack of protocols or to the lack of compliance with present protocols. Oral rinses are frequently used, probably because of the recommendation from various ventilator associated pneumonia prevention bundles.^(1,13-15)^ The great variability in the use of toothbrushes and toothpaste is in alignment with previous studies.^(2,10,12)^

## CONCLUSIONS

Oral health and oral healthcare contribute to an intensive care unit patient's general health, but the intensive care unit staff often finds it difficult to provide such care, primarily because of the absence of training and adequate protocols. The lack of well-established healthcare protocols and training programs leads to the nursing staff's inability to solve oral health problems. The presence of an oral health professional (dentist) to evaluate oral health issues in intensive care unit patients could minimize such problems.

The present study suggests that the presence of a dentist in the intensive care unit routine and implementation of institutional protocols with proper staff training can positively influence attitudes of ICU staff, leading to a more consistent practice of oral healthcare in the intensive care unit.
